# Neoadjuvant Multikinase Inhibitor in Patients With Locally Advanced Unresectable Thyroid Carcinoma

**DOI:** 10.3389/fendo.2019.00712

**Published:** 2019-10-22

**Authors:** Carla Fernanda Nava, Rafael Selbach Scheffel, Ana Patrícia Cristo, Carla Vaz Ferreira, Shana Weber, André Borsatto Zanella, Francisco Costa Paixão, Alceu Migliavaca, José Ricardo Guimarães, Marcia Silveira Graudenz, José Miguel Dora, Ana Luiza Maia

**Affiliations:** ^1^Thyroid Unit, Endocrine Division, Faculdade de Medicina, Hospital de Clínicas de Porto Alegre, Universidade Federal do Rio Grande do Sul, Porto Alegre, Brazil; ^2^Pathology Division, Faculdade de Medicina, Hospital de Clínicas de Porto Alegre, Universidade Federal do Rio Grande do Sul, Porto Alegre, Brazil; ^3^Surgical Division, Faculdade de Medicina, Hospital de Clínicas de Porto Alegre, Universidade Federal do Rio Grande do Sul, Porto Alegre, Brazil

**Keywords:** thyroid carcinoma, multikinase inhibitors, neoadjuvant therapy, unresectable thyroid tumors, locally invasive thyroid tumors

## Abstract

**Background:** Papillary thyroid carcinoma (PTC) is the most common and less aggressive thyroid cancer, but some patients may display locally advanced disease. Therapeutic options are limited in these cases, particularly for those patients with unresectable tumors. Neoadjuvant therapy is not part of the recommended work up.

**Methods:** Report a case of an unresectable grossly locally invasive PTC successfully managed with neoadjuvant therapy and provide a systematic review (SR) using the terms “Neoadjuvant therapy” AND “Thyroid carcinoma.”

**Results:** A 32-year-old man with a 7.8 cm (in the largest dimension) PTC was referred to total thyroidectomy, but tumor resection was not feasible due to extensive local invasion (trachea, esophagus, and adjacent structures). Sorafenib, a multikinase inhibitor (MKI), was initiated; a 70% tumor reduction was observed after 6 months, allowing new surgical intervention and complete resection. Radioactive iodine (RAI) was administered as adjuvant therapy, and whole body scan (WBS) shows uptake on thyroid bed. One-year post-surgery the patient is asymptomatic with a status of disease defined as an incomplete biochemical response. The SR retrieved 123 studies on neoadjuvant therapy use in thyroid carcinoma; of them, 6 were extracted: 4 case reports and 2 observational studies. MKIs were used as neoadjuvant therapy in three clinical cases with 70–84% of tumor reduction allowing surgery.

**Conclusion:** Our findings, along with other reports, suggest that MKIs is an effective neoadjuvant therapy and should be considered as a therapeutic strategy for unresectable grossly locally invasive thyroid carcinomas.

## Introduction

Thyroid carcinoma is the most common endocrine malignancy, accounting for 3.1% of the global incidence of cancers in 2018 (1). Differentiated thyroid carcinoma (DTC), including papillary (PTC) and follicular carcinoma (FTC), is derived from follicular cells of the thyroid gland and accounts for the majority of thyroid malignancies. Approximately 84% of DTC tumors are PTC, affecting mainly female patients aged 40–59 years (2, 3). PTC is usually an indolent tumor, with an excellent prognosis. The initial therapeutic approach to DTC consists of total thyroidectomy, which might be followed by radioactive iodine (RAI) administration, and TSH suppression in selected cases (4–6). The incidence of unresectable PTC is unknown, and for these patients, therapeutic options are limited and disappointing.

Multikinase inhibitors (MKI) are drugs indicated for bulky or rapidly progressing iodine-refractory metastatic DTC, that result in symptomatic or threatening uncontrolled disease not amenable to other therapies (4). The antiproliferative MKI effect has been demonstrated in primary papillary dedifferentiated thyroid cancer (DePTC) cells whereas potent inhibition of angiogenesesis and growth were shown in nude mice bearing thyroid tumor xenografts (7, 8). The antitumor effects were confirmed in clinical studies, and currently, two MKI compounds have been approved by the Food and Drug Administration (FDA) for iodine-refractory PTC treatment: sorafenib and lenvatinib (9, 10). Sorafenib inhibits VEGFR-1, VEGFR-2 and VEGFR-3, RET (including RET/PTC), RAF (including BRAF V600E), and platelet-derived growth factor B receptor. In the DECISION trial, the benefits of sorafenib were evidenced by higher rates of progression-free survival (PFS): 10.8 vs. 5.8 months, *P* < 0.0001. The best response in tumor reduction reached 60%, but no benefit for overall survival was documented (9). Lenvatinib, the second MKI approved, inhibits VEGFRs 1,2, and 3, FGFRs 1,2,3, and 4; PDGFR α, RET, and KIT signaling networks. The SELECT trial showed that patients with locally advanced disease in the lenvatinib group, even those that received another MKI before randomization, achieved a PFS benefit. The median PFS was 18.3 months in the lenvatinib group vs. 3.6 months in the placebo group (HR for progression or death, 0.21; 99% confidence interval, 0.14–0.31; *P* < 0.001). Lenvatinib achieved the best response rate of 64.8% (4 complete responses) vs. 1.5% in the placebo group (*P* < 0.001) (10).

Neoadjuvant therapy refers to the administration of therapeutic agents before a primary treatment, usually surgery, aiming to reduce the size of the tumor (11). The use of MKI as neoadjuvant therapy is a well-established therapeutic tool for several human neoplasias (12–14). However, neoadjuvant treatment is an unusual event in the management of thyroid carcinoma. Indeed, it is not mentioned in the current DTC guidelines (4–6). Here, we report a patient presenting with locally advanced unresectable PTC who displayed a 70% tumor reduction after neoadjuvant treatment with sorafenib, allowing complete surgical resection. Additionally, a systematic review of neoadjuvant therapies for thyroid carcinoma is provided.

## Case Report

### Clinical Presentation and Management

**Figure 1:**
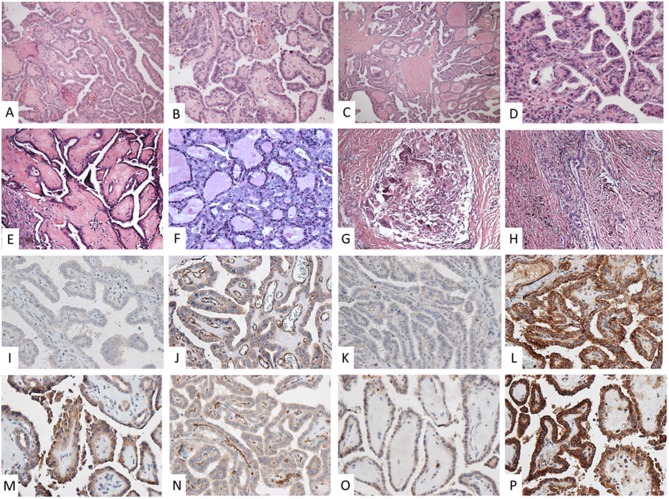
Pre **(A–D)** and post-neoadjuvant **(E–H)** histopathological analysis of the classical papillary thyroid carcinoma surgical specimens. **(A,B)** Complex, branching, randomly oriented papillae with fibrovascular areas (HE 100X); **(C,D)** papillae lined by cuboidal cells, nuclei overlap with finely dispersed optically clear chromatin (HE 400X); **(E)** papillae showing fibrotic fibrovascular areas (HE 400X); **(F)** preserved neoplastic architecture (HE 400X); **(G)** foreign body granuloma (HE 100X); **(H)** densely fibrotic area showing old hemorrhage and residual infiltrative carcinoma (HE 100 X). Expression of VEGF, VEGFR-1, VEGFR2, and CD31 immunolabeling in pre **(I–L)** and post-neoadjuvant **(M–P)** surgical specimens, respectively. **(I)** light positivity in tumor cells and vascular endothelium; **(J)** highlighted a prominent vascular network in the neoplastic papillae; **(K)** negative staining; **(L)** intense staining in tumor cells maintained; **(M,O)** slightly increased positivity in tumor cells; **(P)** intense staining in tumor cells; **(N)** reduction in number and the caliber of vessels within fibrovascular areas (all 400X).

A 32-year-old man presented with a cervical lesion of 7.8 cm (in the largest diameter), which was diagnosed as classical PTC by fine needle aspiration (FNA) cytology analysis and was referred to total thyroidectomy. However, the complete tumor resection was not feasible due to the invasion of trachea, esophagus and adjacent structures, the absence of a clear cleavage plane, along with compression, of cervical vessels, and only a partial resection of the left lobe of the thyroid was performed. The surgical product sent for histopathological examination showed a classical PTC with complex branching randomly oriented papillae with fibrovascular cores lined by cuboidal neoplastic cells. Carcinoma cell nuclei overlapped and showed finely dispersed optically clear chromatin (Figures 1A–D).

**Figure 2:**
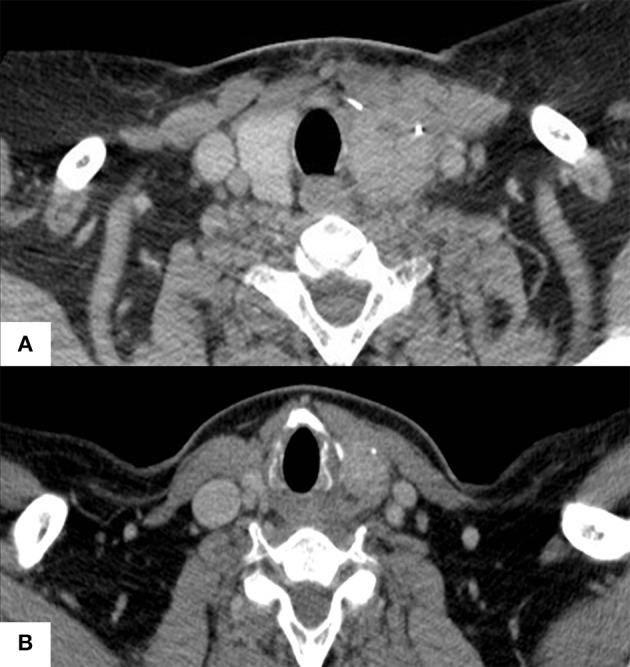
Thyroid CT scans pre **(A)** and after 6 months **(B)** of neoadjuvant sorafenib therapy.

Immediate postoperative computed tomography (CT) displayed a lesion measuring 6.8 × 3.4 × 3.4 cm (Figure 2A) in contact with the carotid space, infiltrating the fat surrounding the vascular structures, with lateral deviation and reduction of the caliber of the internal jugular vein and no cleavage plan with the cervical musculature. Atypical lymph nodes on the left side of the neck up to 1.0 × 1.0 × 1.0 cm in diameter and three nodular lung opacities of 0.6, 0.4, and 0.4 cm diameters, considered non-specific, but suspicious for PTC metastasis were described.

Due to the advanced unresectable disease, neoadjuvant therapy with sorafenib (800 mg per day) was started, aiming to reduce the lesion to allow surgical treatment. During sorafenib use, the patient presented hypertension, and grade II hand-foot syndrome both controlled with oral medications and topical measures. After 6 months of treatment, the large cervical lesion showed a reduction of 70% of the largest diameter from baseline (2.0 × 1.9 × 1.6 cm—Figure 2B) in CT, defined as a partial response by RECIST criteria (15). Now, the lesion showed a cleavage plane with adjacent structures and cervical vessels. Sorafenib was stopped and surgery scheduled for 30 days later. A new CT immediately before surgery documented stable disease (target lesion size: 2.3 × 2.2 × 1.9 cm).

The patient underwent a new surgical procedure, and the entire macroscopic PTC lesion was resected together with four cervical lymph nodes of the left jugular chain and mediastinal fat. Cervical vessels was preserved, but it was necessary to shave the trachea and esophagus and resect the left recurrent laryngeal nerve. The postoperative course was unremarkable, with no need for tracheostomy or ventilatory support, and the patient was discharged home on the third postoperative day. The histopathological analysis demonstrated a 2.5 cm classical PTC and another focus spanning 0.5 cm on the left lobe. Also, a 0.2 cm focus of PTC as identified in adipose tissue, 3 left jugular metastatic lymph nodes and positive surgical margins (pT4a N1b Mx—stage I). Compared to the pre neoadjuvant specimen, residual papillae were fibrotic and extensive areas of stromal fibrosis showing old hemorrhage and foreign body inflammation were observed (Figures 1E–H).

One month after surgery the patient received RAI therapy (100 mCi) administered in a stimulated TSH condition of endogenous hypothyroidism after withdrawing levothyroxine for 4 weeks (TSH 89.8 μUI/mL; normal reference: 0.27–4.2 μUI/mL). Serum thyroglobulin (Tg) showed a value of 42.6 ng/mL (sensibility <0.1 ng/mL) with positive anti-thyroglobulin antibody (AAT) (6.4 UI/mL; reference <4.11 UI/mL). A post-therapy whole body scan (WBS) was performed 10 days after the radioiodine administration, showing uptake at the thyroid bed. The disease status after 1 year of follow-up is an incomplete biochemical response (TSH: 9.1 μUI/mL; Tg: 5.2 ng/mL; AAT: 5.3 UI/mL and imaging exams without evidence of suspicious lesions). A written informed consent was obtained from the patient for the publication of this case report.

### Tumor Markers and Genetic Analysis

Additionally to hematoxylin and eosin staining, immunohistochemistry analysis (IHC) was performed on thin sections (3 μm) of previously formalin-fixed and paraffin-embedded tissues. Expression of VEGF, VEGFR-1, VEGFR2, and CD31 immunolabeling was assessed in pre and post-neoadjuvant surgical specimens (Figures 1I–L). The immunoreactivity was scored as negative (–) absence of labeling; (+) discrete intensity; (++) moderate intensity; and (+++) accentuated intensity. Pre and post-treatment surgical specimens were compared, and the VEGF and VEGFR 1 displayed slightly increased positivity in tumor cells. On the other hand, a reduction in the number and caliber of vessels within fibrovascular cores when analyzing the CD31 antibody was observed (Figures 1M–P).

Molecular DNA analysis was performed in paraffin-embedded tumor tissue using the Magnesil Genomic Fixed Tissue System (Promega, Madison, WI, USA), according to the manufacturer's instructions Point mutations at codon 600 of *BRAF* and at codons 12, 13, and 61 of K-, H-, and N-*RAS* were evaluated by PCR using specific primers and submitted to direct sequencing by standard procedures previously described (16). No known mutation was found in the analyzed exons and isoforms of the RAS gene. The BRAF V600E mutation was also negative.

## Systematic Review

### Methods

The electronic databases PubMed/Medline and Embase were searched for studies of neoadjuvant therapy for thyroid carcinoma. The reference lists of all identified articles were examined, and authors of included studies were consulted to obtain additional information when needed.

Two investigators (CFN and RSS), blinded to each other's rating, independently assessed study eligibility. All data were independently abstracted in duplicate using a standardized abstraction form. Differences in data extraction were resolved by a third party (JMD) and by referencing the original publication.

**Figure 3:**
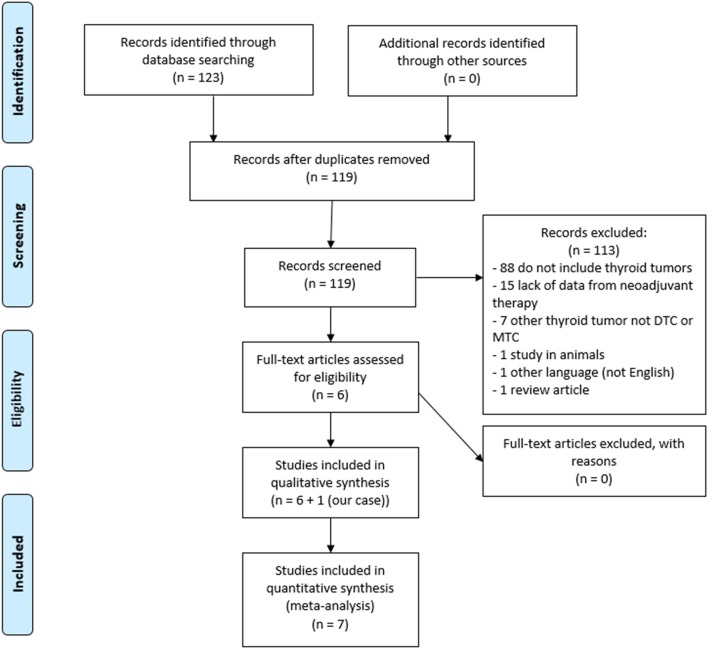
Flow diagram of reference identification and selection.

A search using the terms “Neoadjuvant therapy” AND “Thyroid carcinoma,” with a language filter for articles in English on August 14th, 2018 retrieved 123 articles. Four manuscripts were excluded due to duplicated data. Based on title and abstract content, 88 studies were excluded because they did not include thyroid tumors, 15 because of lack of data from neoadjuvant therapy, 7 because they included thyroid tumors other than DTC or medullary thyroid carcinoma (MTC), 1 was not performed in humans, 1 was written in another language than English (French), and 1 review article. After the inclusion of the present case, a total of 5 case reports and 2 observational studies were included (Figure 3). A summarized description of the characteristics of the studies is shown in Table 1.

**Table 1:** Publications retrieved from the search in database on neoadjuvant therapy and thyroid carcinoma.

**Author**	**Country/Year**	**Type of study/Tumor histology**	**Number of patients**	**Age (years or mean age)**	**Neoadjuvant therapy**	**Response to therapy**	**Surgery**	**Disease status**
Besic et al.	Slovenia/2012	Retrospective/Follicular or Hurthle cell	29	61	Vinblastine (19 patients); vinblastine + adriamycin (5 patients); or other ChT (5 patients)	45% patients with >50% tumor reduction	15 patients no residual tumor; 10 patients microscopic residual tumor; and 4 patients macroscopic residual tumor	4 excellent response 2 with persistent disease 17 deaths from the disease and 6 deaths from other causes
Besic et al.	Slovenia/2013	Retrospective/PTC and variants^*^	16	63	Vinblastine (11 patients); vinblastine + adriamycin (2 patients); or other ChT (3 patients). Of note, 4 patients received neoadjuvant RhT due to tumor progression	44% patients with >50% tumor reduction	2 patients no residual tumor; 10 patients microscopic residual tumor; and 4 patients macroscopic residual tumor	2 excellent response 4 with persistent disease 8 deaths from the disease
Shingu et al.	Japan/1998	Case report/PTC	1	57	RAI (180 mCI)	50% of tumor reduction	Total thyroidectomy, partial resection of trachea, bilateral modified neck dissection, and upper mediastinal dissection	Alive for 1 year
Cleary et al.	USA/2010	Case report/MTC	1	45	RhT + Cisplatin plus adriamycin after carboplatin plus placlitaxel and last sunitinib for 19	Tumor reduction allowing surgical resection	Total thyroidectomy, resection of the mediastinal mass, and a central compartment and left neck dissection	Alive; calcitonin 106 pg/mL, without evidence of macroscopic disease
**Multikinase Inhibitors as Neoadjuvant Therapy**								
Tsuboi et al.	Japan/2017	Case report/PTC	1	73	Lenvatinib for 18 weeks	84% of tumor reduction	Total thyroidectomy, modified neck dissection, resection of the muscular layer of the esophagus, and a tracheal sleeve resection and reconstruction	Alive without distant metastases
Danilovic et al.	Brazil/2018	Case report/PTC	1	20	Sorafenib for 13 months	Tumor reduction that allowed surgical therapy	Near total thyroidectomy and bilateral neck dissection	Structural incomplete response
Nava et al.	Brazil/2019	Case report/PTC	1	32	Sorafenib for 6 months	70% tumor reduction	Total thyroidectomy, left jugular chain dissection, shaving of trachea, and esophagus	Biochemical incomplete response

ChT, chemotherapy; RhT, radiotherapy; PTC, papillary thyroid carcinoma; MTC, medullary thyroid carcinoma; RAI, radioiodine therapy; NA, not available; ^*^poorly differentiated PTC; Classical PTC; Follicular variant of PTC; and Hurthle cell variant of PTC.

This systematic review was planned and reported according to the Preferred Reporting Items for Systematic Reviews and Meta-Analyses (PRISMA) statement guidelines (17).

## Results

### Chemotherapy

Two retrospective studies of neoadjuvant chemotherapy for patients with DTC considered inoperable were identified (18, 19). Treatment regimens consisted mostly of vinblastine, vinblastine plus adriamycin or other regimens in the two studies. Four follicular thyroid carcinomas (FTC) of 16 patients additionally received radiotherapy due to tumor progression despite chemotherapy and before surgery. Treatment response results were similar between PTC and FTC populations: 13/29 (45%) and 7/16 (44%) of patients with a tumor reduction of more than 50%, respectively. All patients underwent surgery, allowing a total or near total thyroidectomy in 24/29 (83%) and 11/16 (69%). After surgery, radioiodine was administered to 26/29 (90%) and 14/16 (87%); and radiotherapy to 20/29 (69%) and 12/16 (75%), respectively. In the follow-up 4/29 (14%) and 2/16 (13%) patients were disease-free; 2/29 (7%) and 4/16 (25%) had persistent disease and 17/29 (59%) and 8/16 (50%) died from the disease, respectively. Mean disease-free survival was 110 months (7–201) for PTC and 110 months (5–218) for FTC patients.

### Radioiodine

We identified one case report on the use of RAI as neoadjuvant therapy for PTC. A 57-year-old woman, with inoperable PTC, tumor size of 6.0 cm with symptoms of hoarseness and dyspnea. the patient received 3 doses of RAI from March 1995 to November 1996, with a cumulative radioiodine activity of 270 mCi. At this time, the tumor diameter was 3.0 cm, and total thyroidectomy was performed 30 days after the last RAI dosing; the patient was followed up for 1 year, but the final disease status was not reported (20).

### Multikinase Inhibitors

Three previous case reports of neoadjuvant MKI for thyroid carcinoma were identified. The first report describes a 45-year-old woman with suspected anaplastic thyroid carcinoma who initially received radiotherapy and chemotherapy (cisplatin and doxorubicin) for 5 weeks. As no response was observed, the chemotherapy was shifted to carboplatin and paclitaxel for 2 weeks, still in association with radiotherapy. Due to the absence of response, sunitinib 50 mg daily was initiated in an intermittent schedule (4 weeks on and 2 weeks off medication). Total thyroidectomy was performed after 19 months of therapy when the tumor was considered potentially resectable. The histopathological analysis revealed MTC (21).

The second case was a 73-year-old man with a locally advanced PTC for whom surgery was considered highly invasive. He received lenvatinib 14 mg/day for 18 weeks, resulting in a primary tumor reduction of 84%, along with a 56% reduction of cervical lymph nodes metastases. Total thyroidectomy was then performed, preserving the patient's esophagus. The patient received radioiodine and did not present evidence of distant metastases at 10 months follow-up after surgery (22).

The third report was a 20-year-old man with symptomatic PTC. Surgery was attempted but was stopped due to transoperative profuse tumor bleeding. At that time, sorafenib 800 mg daily was started and maintained for 13 months. The tumor displayed a significant reduction, which allowed surgery. Due to the tracheal invasion, the patient underwent cervical external beam radiotherapy and radioiodine dosing (the total activity of 300 mCi). At 52 months of follow-up, the patient has stable, persistent structural disease (23).

## Discussion

PTC is usually an indolent tumor with an excellent prognosis. However, a small but significant percentage of patients with thyroid carcinomas may have locally advanced disease at initial presentation. Differentiated thyroid cancer (DTC) with Invasion of surrounding structures can lead to morbid procedures such as laryngectomy and tracheal resection. Invasion of trachea or larynx occur in 3.6–22.9% of thyroid cancer patients and advanced airway invasion with endoluminal tracheal involvement occurs in 0.5–1.5% of cases (24–26). Due to the disappointing results obtained with previous chemotherapy strategies, the current DTC guidelines do not recommend neoadjuvant therapy for patients with unresectable in whom surgery is contraindicated (4).

Indeed, a systematic literature search identified only two observational, retrospective studies of neoadjuvant chemotherapy for patients with inoperable thyroid carcinomas, both from the same Slovenian group (18, 19). Of note, 71% (*n* = 32/45) of these patients required complementary RAI administration, and there is no report on the side effects. These findings limit the inferences about the risk/benefit balance of neoadjuvant chemotherapy for thyroid carcinoma. RAI administration was used as neoadvujant therapy in one case report, which limits conclusions.

MKI has only recently emerged as an alternative treatment for refractory-DTC thyroid cancer. However, it has rarely considered as a potential neoadjuvant therapy strategy for thyroid cancer. Here, we describe a case of a young male patient with unresectable grossly locally invasive PTC successfully managed with MKI neoadjuvant therapy. After MKI therapy, the patient underwent surgical resection and RAI administration and currently presents an incomplete biochemical status. A literature search showed the use of MKI as neoadjuvant therapy for locally advanced DTC in 4 case reports; Tumor reduction was observed in all cases, allowing posterior surgery, three of them with complete resection. These data point out MKIs as an effective and feasible alternative for unresectable locally advanced thyroid tumors, potentially changing the course of the disease in those patients with advanced local disease.

An original aspect of this study was the opportunity to evaluate the histopathological changes secondary to MKI therapy in a PTC specimen. Gross pathological examination showed a significant reduction in the tumor size while microscopic examination revealed a fibrotic and hemorrhagic residual tumor. Changes in VEGF and VEGFR immunohistochemical staining, together with decreases of the CD31-positive intratumoral vascular network, corroborate the powerful antiangiogenic and antiproliferative effect of sorafenib on thyroid tumor cells. Of interest, Yuen et al. described similar findings in a renal cell carcinoma xenograft model where sorafenib-treated mice showed reduced mean percentage of CD-31-positive endothelial cells (27).

In summary, the use of MKI neoadjuvant therapy is particularly beneficial for locally advanced thyroid carcinomas. Our observations shed light on new treatment strategies, which can potentially minimize morbidity, improving disease status and long-term prognosis in a subgroup of patients with locally aggressive thyroid carcinoma.

## Data Availability Statement

The datasets generated for this study are available on request to the corresponding author.

## Ethics Statement

Ethical review and approval was not required for the study on human participants in accordance with the local legislation and institutional requirements. The patients/participants provided their written informed consent to participate in this study.

## Author Contributions

CN, RS, JD, and ALM contributed to the study conception, design, data analysis, interpretation, and manuscript preparation. CN, AC, CF, SW, AZ, FP, AM, MG, JG, and ALM were responsible for data collection, data analysis, and manuscript preparation. All authors read and approved the final manuscript version.

### Conflict of Interest

ALM has served as an advisor/speaker for Sanofi-Genzyme within the past 2 years. ALM and CF have served as principal investigator and coordinator, respectively, in multicenter studies for Astra-Zeneca and Sanofi-Genzyme within the past 2 years. The remaining authors declare that the research was conducted in the absence of any commercial or financial relationships that could be construed as a potential conflict of interest.
